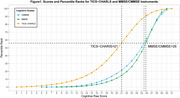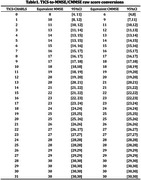# Crosswalk Study of cognitive screening tests between two Chinese cohorts

**DOI:** 10.1002/alz70860_101929

**Published:** 2025-12-23

**Authors:** Mingyue Gao, Ruixin Wang, Ya Fang

**Affiliations:** ^1^ Health and Aging Research Center, School of Public Health, Xiamen University, Xiamen, China, Xiamen, Fujian, China; ^2^ National Institute for Data Science in Health and Medicine, Xiamen University, Xiamen, Fujian, China

## Abstract

**Background:**

The use of different cognitive scales poses a big challenge for direct cross‐study or cross‐national comparisons of cognition. To facilitate further comparisons and data integration, we developed a crosswalk that harmonizes cognitive scales utilized in two Chinese nationally representative cohorts.

**Method:**

Three cognitive scales were utilized: the Telephone Interview for Cognitive Status (TICS‐CHARLS), the Mini‐Mental State Examination (MMSE), and its Chinese‐modified version (CMMSE). The study sample comprised elderly adults aged 60 years and older from the 2018 China Health and Retirement Longitudinal Study (CHARLS). We further divided the sample into two groups: 3,396 participants assessed both TICS‐CHARLS and MMSE, and 4,811 participants assessed both TICS‐CHARLS and CMMSE. Each study sample was split into training (70%) and validation (30%) subgroups. Correlation coefficients, classification agreements, and percentile ranks were analyzed between scales. Crosswalks were developed using equipercentile equating with log‐linear smoothing and were further validated through Bland‐Altman plots.

**Result:**

TICS‐CHARLS demonstrated strong correlations with both MMSE (=0.659, *p* <0.001) and CMMSE (=0.729, *p* <0.001). The screening concordance of normal or impaired cognition classifications were 86.54% and 83.66% between TICS‐CHARLS and MMSE/CMMSE, respectively.

Two conversion tables, TICS‐CHRALS to MMSE/CMMSE, were developed with robust performance. For example, a TICS‐CHARLS score of 21 is equivalent to both an MMSE score and a CMMSE score of 26, with all these scores falling around the same percentile rank of 56%. The conversion tables demonstrated high prediction precision, with narrow confidence intervals (two points or less), except relatively wider confidence intervals for sparse data points at the lower end of distribution. The conversion from TICS‐CHARLS to CMMSE demonstrated better precision than that to MMSE. Notably, when TICS‐CHARLS scored 14 points or higher, TICS‐CHARLS equated to the same scores for both MMSE and CMMSE. Validation analyses indicated good agreement between converted and raw scores, with over 80% of converted scores falling within ±3 points of raw scores.

**Conclusion:**

We developed conversion tables and figures to facilitate a direct crosswalk between TICS‐CHARLS and MMSE/CMMSE scores. The crosswalk demonstrated good accuracy, precision, and validity. The conversion will enable cognitive data integration and comparison across two cohorts.